# DNGR-1, a Dendritic Cell-Specific Sensor of Tissue Damage That Dually Modulates Immunity and Inflammation

**DOI:** 10.3389/fimmu.2019.03146

**Published:** 2020-02-07

**Authors:** Francisco J. Cueto, Carlos del Fresno, David Sancho

**Affiliations:** Laboratory of Immunobiology, Centro Nacional de Investigaciones Cardiovasculares, Madrid, Spain

**Keywords:** C-type lectin receptor, dendritic cells, immunity, inflammation, DNGR-1, Clec9a, cross-presentation

## Abstract

DNGR-1 (encoded by *CLEC9A*) is a C-type lectin receptor (CLR) with an expression profile that is mainly restricted to type 1 conventional dendritic cells (cDC1s) both in mice and humans. This delimited expression pattern makes it appropriate for defining a cDC1 signature and for therapeutic targeting of this population, promoting immunity in mouse models. Functionally, DNGR-1 binds F-actin, which is confined within the intracellular space in healthy cells, but is exposed when plasma membrane integrity is compromised, as happens in necrosis. Upon F-actin binding, DNGR-1 signals through SYK and mediates cross-presentation of dead cell-associated antigens. Cross-presentation to CD8^+^ T cells promoted by DNGR-1 during viral infections is key for cross-priming tissue-resident memory precursors in the lymph node. However, in contrast to other closely related CLRs such as Dectin-1, DNGR-1 does not activate NFκB. Instead, recent findings show that DNGR-1 can activate SHP-1 to limit inflammation triggered by heterologous receptors, which results in reduced production of inflammatory chemokines and neutrophil recruitment into damaged tissues in both sterile and infectious processes. Hence, DNGR-1 reduces immunopathology associated with tissue damage, promoting disease tolerance to safeguard tissue integrity. How DNGR-1 signals are conditioned by the microenvironment and the detailed molecular mechanisms underlying DNGR-1 function have not been elucidated. Here, we review the expression pattern and structural features of DNGR-1, and the biological relevance of the detection of tissue damage through this CLR.

## Introduction

Cell death is a complex and diverse process that occurs in both homeostatic and disease conditions (1). The immune system has developed mechanisms to distinguish different types of cell death by the detection of molecular cues termed damage-associated molecular patterns (DAMPs) that help to modulate immunity and maintain homeostasis (1, 2). Sensing diverse forms of cell death may result in tolerogenic or immunogenic responses, depending on the DAMP signal(s) and the existence of concomitant pathogen-associated molecular patterns (PAMPs) (1). Filamentous actin (F-actin) is the major component of the actin cytoskeleton, which is normally concealed inside the cytoplasm. However, upon loss of plasma membrane integrity, F-actin can be sensed by immune cells and acts as a DAMP (3–5). DNGR-1 (*CLEC9A*) is a dendritic cell (DC)-specific receptor that senses polymeric F-actin exposed during necrotic cell death (3, 5, 6) and favors cross-presentation of antigens in the necrotic cargo (7–9). Yet, sensing of cell death via DNGR-1 also modulates neutrophil infiltration, thus limiting inflammation. In this setting, detection of tissue damage through DNGR-1 acts as a negative feedback loop to limit immunopathology (6). Here, we compile the current knowledge about DNGR-1 and its function in homeostasis and disease.

## DNGR-1 as a Targetable cDC1 Marker

DNGR-1 is a C-type lectin receptor (CLR) encoded by the *CLEC9A* gene (10, 11), which is located in the NK complex on human chromosome 12 (12) and mouse chromosome 6 (10). In the mouse, *Clec9a* expression is highly restricted to type 1 conventional DCs (cDC1s) (10–13), common DC progenitors (CDP), and pre-DC progenitors (14), and it is also expressed at low levels in plasmacytoid DCs (pDCs) (10, 13, 14). DNGR-1 is, however, not expressed by human pDCs and is highly restricted to human cDC1s in blood (11, 15, 16), lymphoid organs (16, 17), and peripheral tissues (16), including tumors (18, 19). Of note, in a mouse model that allows genetic tracing using a fluorescent protein that is stably expressed upon DNGR-1 expression (*Clec9a*-Cre), almost exclusively DCs are labeled, with some signal also among populations traditionally thought to represent red pulp macrophages and lung and kidney CD64^+^ cells that might derive from DC precursors (14, 20).

The restricted expression profile of DNGR-1 may be targeted for biomedical purposes (summarized in Table 1). Targeting of antigens to DNGR-1 using antibodies allows for specific delivery to cDC1s (10, 13, 21), which excel at cross-presentation (22), resulting in improved cross-priming of CD8^+^ T cells when administered under the cover of an adjuvant (10). Alternatively, specific peptides selected by *in vitro* evolution systems and beads coated with a synthetic F-actin/myosin II complex can be used for *in vivo* targeting of antigens toward DNGR-1 (23, 24). Moreover, anti-DNGR-1 antibodies can be used to deliver antigen-containing nanoemulsions with immunostimulatory properties (25). Upon engagement with F-actin or monoclonal antibodies, surface DNGR-1 is internalized (10) and directed to non-degradative endosomal compartments (8), which are associated with enhanced antigen cross-presentation (26).

**Table 1:** Main features and potential biomedical applications of DNGR-1/*CLEC9A*.

**DNGR-1/*CLEC9A* feature**	**Purpose**	**Strategy**	**Biomedical application**
DC-restricted expression profile	Prediction of cDC1 infiltration from bulk transcriptomics (cDC1 signature)	RNA sequencing	Prediction of overall survival in some cancers Potential application to diseases where cDC1 may play a key role
	Targeted delivery of antigens, adjuvants, cytokines, or drugs to cDC1s	Anti-DNGR-1 antibodies Synthetic DNGR-1 ligands DNGR-1-specific peptides DNGR-1-specific aptamers Functionalized nanoparticles	Induction of immunity or tolerance by targeting antigen to cDC1s
Induction of cross-presentation	Promoting retention of cargo in cross-presenting compartments	Synthetic DNGR-1 ligands	Immunization Tolerance?
Control of inflammation	Reducing neutrophil infiltration	Synthetic DNGR-1 ligands	Dampening inflammation?
	Increasing neutrophil infiltration	DNGR-1 blockade	Increasing tissue repair? Increasing response to specific infections?

DNGR-1-targeted delivery of antigen can also promote MHC-II antigen presentation to CD4^+^ T cells (13, 21). Adjuvant-free administration of DNGR-1-targeted antigens promotes proliferation and generation of antigen-specific regulatory CD4^+^ T cells, while coadministration of poly(I:C) (TLR3 ligand) or curdlan (Dectin-1 ligand) favors the generation of antigen-specific Th1 or Th17 cells, respectively (27). In accordance with these data, human BDCA3^+^ cDC1s loaded with keyhole limpet hemocyanin (KLH) antigen in the presence of poly(I:C) and R848 (TLR7/8 ligand) induce the proliferation and IFNγ production of KLH-responsive CD4^+^ T cells (28). Additionally, antigen targeting to DNGR-1 induces durable humoral responses (13, 29, 30) through the induction of antigen-specific T follicular helper cells (31). In fact, the generation of T follicular helper cells and humoral responses against anti-DNGR-1-coupled OVA does not require adjuvants (31). To highlight the translational potential of these findings, type I IFN has been targeted to cDC1s both *in vitro* and in humanized mice with the purpose of modulating cancer and autoimmunity (32, 33).

The specific expression pattern of *Clec9a*, particularly in humans, can also be used as a specific signature to track cDC1s (Table 1). For instance, the *Clec9a* promoter has been used as a reporter system for the reprogramming of fibroblasts into the cDC1 lineage with the transcription factors PU.1, IRF8, and BATF3 (34). This is of relevance in cancer immunology, where cDC1 infiltration within solid tumors has been associated with effective antitumor immunity and prognosis of increased overall survival in a variety of tumor types (35–37). Single-cell RNA sequencing has allowed for the identification of immune cell populations in cancer, both at the tumor bed (38) and its draining lymph node (17), where *Clec9a* expression is even more restricted to cDC1s (17, 38). Thus, expression of *CLEC9A* alone within resected tumors, as a marker of cDC1s, constitutes a great prognostic factor for cancer patients (39) and could be used to select therapeutic strategies (18).

## DNGR-1 Structure and Ligand Binding

DNGR-1 is a type II membrane receptor that belongs to group V of CLRs, bearing a single C-type lectin-like domain (CTLD) linked by a neck region to a transmembrane domain followed by an intracellular domain containing a hemi-immunoreceptor tyrosine–based activation motif (hemITAM) signaling motif (7, 12), crucial for downstream signaling (10, 12). DNGR-1 is a glycosylated protein, as indicated by the increased electrophoretic motility of DNGR-1 after PNGase F treatment (40). Thus, DNGR-1 results in two bands in western blot, because it can form different glycoforms. PNGase F treatment does not reduce the number of observed bands, which suggests that the bands relate to O-glycosylation or α(1-3)-fucosylation variants (40).

The neck region contains a cysteine residue that allows for dimerization of the receptor, a key feature in DNGR-1 function (40). That cysteine residue is located at positions 94 and 96 in mouse and human, respectively (10, 12, 40). Moreover, the neck region of DNGR-1 allows for the formation of reduction-resistant homodimers in low pH or ionic strength solutions, which can be reversed by increasing the pH or the ionic strength of the medium (40). The formation of reduction-resistant homodimers of DNGR-1 relies on changes in the tertiary structure of the protein and requires the neck segment Q95–S104 (40). However, another segment of this neck region (N81–T90) destabilizes the formation of those homodimers, as its elimination or substitution by alanine residues results in the stabilization of homodimers, regardless of the redox conditions (40).

The CTLD of DNGR-1 does not contain the classical calcium-dependent carbohydrate binding domain. The crystal structure of the CTLD of human DNGR-1 (residues S111-L236) has been solved (3). The E202-N208 segment of DNGR-1 CTLD could not be resolved in this crystal, suggesting that it could behave as a flexible region (3). However, some caution is needed as some regions bound by anti-DNGR-1 antibodies are predicted to be buried in the structure in this model (41).

F-actin, which is exposed on necrotic cells upon loss of membrane integrity, is the only DNGR-1 ligand identified so far (3, 5). F-actin seems like an ancestral molecular cue for cell death, as DNGR-1 can recognize metazoan F-actin from yeasts (3, 5). Electron cryomicroscopy of F-actin complexed with the dimeric extracellular domain of DNGR-1 shows that, in fact, only one subunit of DNGR-1 dimers is stabilized on a site on F-actin that involves three actin subunits: two consecutive subunits from the same protofilament and one from the neighboring protofilament (4). Of note, myosin II, an F-actin-associated motor protein, potentiates the binding of dimeric DNGR-1 to F-actin, explaining why F-actin from cell lysates binds more efficiently to DNGR-1 dimers than *in vitro* polymerized F-actin (24). A likely explanation for the increased affinity may be the spatial disposition of F-actin filaments promoted by myosin II, which might facilitate the engagement of both DNGR-1 dimer subunits to adjacent, aligned F-actin filaments. However, other actin-binding proteins, such as spectrin or troponin, do not alter the binding of DNGR-1 to F-actin (24) contrary to what had been previously suggested (3).

## Induction of CD8^+^ T Cell Responses Against Dead Cell-Associated Antigens

Ablation of DNGR-1 compromises cross-priming of CD8^+^ T cells against dead-cell associated antigens (7), but not against soluble or bead-bound antigen (7, 8). However, DNGR-1 engagement does not induce activation hallmarks in cDC1s, such as costimulatory molecule expression or cytokine production (7–9). Moreover, DNGR-1 does not function as a scavenger receptor (7). DNGR-1 rather controls necrotic cargo compartmentalization (8). Diversion of cargo to non-lysosomal, non-acidic, non-degradative endosomal compartments promotes CD8^+^ T cell cross-priming (26, 42). Accordingly, inhibitors of lysosomal function restore the cross-presentation capacity of DNGR-1-deficient cDC1s (9).

Following vaccinia virus (VACV) or herpes simplex virus (HSV)-1 infection, DNGR-1 controls the cross-presentation of viral antigens, but since viral antigens can be also directly presented, the global CD8^+^ T effector response is not affected in DNGR-1-deficient mice (8, 9, 43–45). However, DNGR-1-mediated cross-priming is key for the formation of T resident memory (Trm) precursors, as transfer of CD8^+^ T cells primed in the draining lymph node of DNGR-1-deficient mice in the first 72 h after VACV infection to WT recipients results in deficient generation of Trm (45). Consistent with this, administration of DNGR-1-blocking antibodies to WT mice during the priming phase of VACV infection (first 72 h) impairs the formation of Trm, but not at later time points (45). Cross-priming by cDC1s favors the retention of CD8^+^ T cells being primed at lymph nodes in the early infection phase (45). Accordingly, the administration of an inhibitor of CD8^+^ T cell lymph node egress, FTY720, increases the generation of Trm in DNGR-1-deficient mice. Interestingly, it does so also in WT mice (45), which could be used to improve current vaccination strategies. In this regard, DNGR-1 is needed for generating Trm specific for influenza virus NP protein, which might be applicable in the design of broadly protective flu vaccines (45) (Table 1).

## Modulation of Inflammation by DNGR-1

In addition to the long-known role for DNGR-1 in the generation of CD8^+^ T cell responses against dead cell-associated antigens (7), a new role has been uncovered for DNGR-1 in innate immunity (6). DNGR-1-deficient mice display an exacerbated neutrophil recruitment during caerulein-induced acute pancreatitis (6). Neutrophils, which participate in the defense of the organism against infectious diseases, also generate tissue damage, a phenomenon called immunopathology (46–48). DNGR-1 limits the recruitment of neutrophils to sterile injured tissue, thus acting as negative feedback on tissue damage. Of note, the effect of DNGR-1 is also observed in *Rag1*-deficient mice lacking T and B cells (6), indicating that this observation is independent of the previously described role of DNGR-1 in cross-presenting antigen (7). Immunopathology can also worsen the outcome of infectious processes (49, 50). Similar to its role in the caerulein model, DNGR-1 limits infiltration of neutrophils and tissue damage in kidneys from *Candida albicans*–infected mice, reducing morbidity and mortality (6). Once again, this process occurs independently of the adaptive immune compartment (6). Indeed, neutrophils are key mediators of increased immunopathology in DNGR-1-deficient mice, as depletion of neutrophils in these two models of disease prevents the increased tissue damage (6).

The administration of anti-DNGR-1 blocking antibodies recapitulates the increased neutrophil infiltration in WT but not in *Batf3*-deficient mice, which indicates that cDC1s are fundamental drivers of this phenotype (6). These results highlight the relevance of *Batf3*-dependent cDC1s in the regulation of neutrophil recruitment, which was corroborated in different models of bacterial infections in the skin, where a subset of activated cDC1s has been identified as responsible for driving neutrophil infiltration (51).

As indicated before, DNGR-1 does not induce cytokine production in cDC1s (7, 8). However, the engagement of DNGR-1 with a synthetic DNGR-1 ligand containing F-actin/myosin II (24) limits the induction of pro-inflammatory genes *Tnf*, *Cxcl2*, and *Egr2* on cDC1s in response to whole glucan particles (WGP), a Dectin-1 ligand that mimics *C. albicans* recognition (6). These results indicate that DNGR-1 may specifically dampen heterologous signaling pathways. Indeed, the capacity of CLRs to cross-talk with heterologous receptors is well-known (52, 53). As CXCL2 is one of the main drivers of neutrophil attraction to inflammation sites upon *C. albicans* infection (54, 55), administration of pepducin, a peptide that blocks signal transduction via CXCR2, the main receptor for CXCL2, reverts the increased neutrophil recruitment in DNGR-1-deficient mice (6). A series of bone-marrow chimera experiments demonstrated that *Cxcl2* expression by cDC1s is needed for the exacerbated neutrophilia in the absence of DNGR-1 (6).

These results illustrate that, in addition to its contribution to adaptive immunity by promoting CD8^+^ T cell cross-priming, DNGR-1 contributes to the regulation of innate immunity by cDC1s by harnessing inflammatory cues that control the influx of neutrophils into damaged tissues. Whether the regulatory role of DNGR-1 applies only to neutrophils or may affect other cells depending on the origin and tissue location of inflammation, and its potential biomedical applications (Table 1) deserves further investigation.

## The Signaling Crossroads at DNGR-1

The intracellular segment of DNGR-1 features a hemITAM, which resembles the signaling core of Dectin-1 (7, 12). Upon engagement by agonist ligands, the hemITAM of Dectin-1 becomes phosphorylated and recruits the kinase SYK to promote myeloid cell activation (56, 57). Recognition of dead cells by DNGR-1 also recruits SYK (7), which is necessary for the cross-presentation of dead cell-associated antigens (9, 45). However, as indicated above, DNGR-1 does not mediate activation of DCs (8). The difference between Dectin-1 and DNGR-1 can be attributed to their intracellular signaling motif, as chimeric Dectin-1 containing the hemITAM of DNGR-1 does not promote myeloid cell activation upon agonist engagement (8). The capacity of Dectin-1 to induce cytokine production has been attributed to a DEDG sequence preceding its hemITAM. However, DNGR-1's equivalent sequence is AEEI. Thus, Dectin-1 chimeric receptors with an I6G mutated DNGR-1 hemITAM induce activation, while those bearing A3D do not, indicating that the I6 residue is central to the difference between DNGR-1 and Dectin-1 hemITAMs (8).

The recent finding that DNGR-1 can dampen cDC1-driven inflammation sheds light into a new signaling modality for DNGR-1 (6). Here, engagement of DNGR-1 by its ligand is accompanied by phosphorylation of the phosphatase SHP-1, which limits signaling through heterologous receptors such as Dectin-1, resulting in reduced NFκB signaling and PLCγ2 phosphorylation (6). Therefore, mice lacking DNGR-1 display exacerbated production of CXCL2 and other mediators of inflammation when these heterologous receptors are triggered (6). *In vitro*, the synthetic ligand of DNGR-1 inhibits the expression of *Cxcl2* by cDC1s activated with WGP, but the administration of SHP-1 inhibitors reverts this inhibition, reinforcing the role of the phosphatase in this context (6). Accordingly, mice that lack SHP-1 in the CD11c compartment also display increased neutrophil infiltration in their kidneys upon *C. albicans* infection, mimicking DNGR-1 deficiency (6). These results are in line with the described role of the SHP-1 phosphatase in shifting ITAM-containing receptors, such as FcγRIIA (58) and Mincle (59), from an activating to an inhibitory configuration.

## Perspective

**Figure 1:**
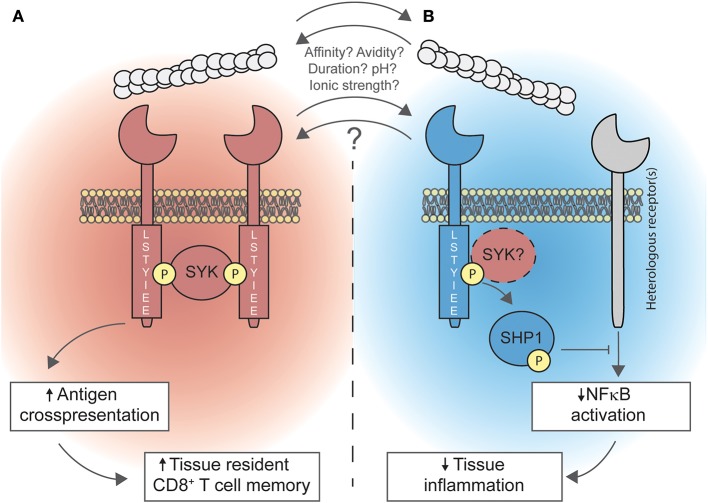
Duality of DNGR-1 in promoting cross-presentation and limiting inflammation. **(A)** Upon recognition of F-actin, DNGR-1 signals through the spleen tyrosine kinase (SYK). Signaling of DNGR-1 through SYK diverts phagocytosed cargo toward non-degradative, non-acidic, non-lysosomal compartments. This is a key step for cross-presenting dead cell-associated antigens, which is necessary for the optimal generation of resident memory CD8^+^ T cells in peripheral tissues. **(B)** Also, DNGR-1 may activate the SH2 domain-containing phosphatase 1 (SHP-1), through a mechanism that might involve SYK, dampening NFκB activation triggered by heterologous receptors; this process limits tissue inflammation by dampening the recruitment of neutrophils to inflammatory foci. Whether both processes occur simultaneously or whether other factors (such as ligand affinity or avidity, ligand binding duration, environmental pH, or ionic strength) determine the signaling modality of DNGR-1 is not known yet.

The expression profile of DNGR-1 and the capacity of the receptor to recognize F-actin, which acts as a DAMP, have been well-characterized. Tissue damage sensing may result in both immunity or disease tolerance (1), and DNGR-1 has been implicated in both processes (Figure 1), but many questions remain open surrounding this receptor. For instance, the expression of DNGR-1 in DC progenitors (14) is intriguing. What would be the role of this tissue damage receptor in DC ontogeny and development? How would DNGR-1 sense tissue damage in this context or are there some other yet-unknown DNGR-1 ligands?

Cross-presentation by cDC1s has been highlighted as a key element in the antitumor immune response (18, 22, 60–62), and the abundant necrosis that occurs within tumors (63, 64) leads to the hypothesis that DNGR-1 might play a role in promoting antitumor immunity (8). While DNGR-1 can be targeted with antigens bound to antibodies, directed peptides, or F-actin/myosin-coated beads to vaccinate against tumors (10, 23, 24), the *in vivo* role of DNGR-1 in cancer immunity has not been addressed yet.

Another aspect of DNGR-1-mediated cross-presentation that may need further analysis is whether DNGR-1 can promote tolerance to cross-presented antigens, a phenomenon called cross-tolerance. To generate cross-tolerance, tissue-specific antigens are continuously presented by DCs, resulting in proliferation of dysfunctional and short-lived CD8^+^ T cells (65). DNGR-1 does not play a role in cross-tolerance of OT-I T cells transferred into RIP-OVA mice, which express membrane-bound OVA under the control of the rat insulin promoter (8). However, whether this model is ideal for testing cross-tolerance to necrosis-derived antigens is arguable, since the source of antigen is likely apoptotic rather than necrotic cells. Thus, the function of DNGR-1 in models for cross-tolerance in the presence of necrosis should be evaluated.

Part of the molecular requirements for the interaction of DNGR-1 with SYK and the SYK-mediated outcomes of DNGR-1 engagement by its ligands have been elucidated (40). However, the molecular mechanisms by which DNGR-1 controls cross-presentation of necrotic cargo remain obscure. Regarding the role of DNGR-1 in inflammation, the particular requirements (e.g., ligand affinity or avidity) that regulate the binding of SYK or SHP-1 to DNGR-1 hemITAM remain unexplored. In fact, as for other receptors that signal through the inhibitory ITAM configuration, transient SYK binding and hypophosphorylation of the hemITAM may be required for SHP-1 association (58, 59).

DNGR-1 forms homodimers due to a cysteine in its neck region, which can become resistant to reduction under conditions of low pH or low ionic strength. DNGR-1 dimerization allows for more efficient binding of the multivalent ligand, particularly when F-actin is cross-linked by myosin II (24). The relevance of this tertiary conformation has been associated with the role of DNGR-1 in antigen handling within phagocytic compartments, where pH drops. However, the extracellular medium may suffer changes in pH or ionic strength in certain situations, such as infection, ischemia, or cancer (66). Also, whether the alternative conformations of DNGR-1 impact its signaling modality remains unexplored.

Overall, the versatility of DNGR-1 promoting cross-priming or restraining inflammation in different scenarios has been reported. Additional studies should shed further light on the relevance of this receptor in clinical settings where necrosis represents a relevant feature, such as infection, autoimmunity, or cancer.

## Author Contributions

FC prepared the figure and conceptualized and wrote the manuscript. CF conceptualized and edited the manuscript. DS contributed to funding acquisition and supervised, conceptualized, and wrote the manuscript.

### Conflict of Interest

The authors declare that the research was conducted in the absence of any commercial or financial relationships that could be construed as a potential conflict of interest.
